# Floating-Variant Medial Elbow Dislocation: A New Classification System

**DOI:** 10.7759/cureus.30200

**Published:** 2022-10-11

**Authors:** Mohamad K Moussa, Ali H Alayane, Johnny Abboud, Ahmad A Abed Ali, Mohammad O Boushnak

**Affiliations:** 1 Orthopedic Surgery, Lebanese University, Faculty of Medical Sciences, Beirut, LBN; 2 Orthopedic Surgery and Traumatology, Hôpitaux Civils de Colmar, University of Strasbourg, Faculty of Medicine, Strasbourg, FRA; 3 Orthopedic Surgery, Université Libre de Bruxelles (ULB) - Erasme University Hospital, Brussels, BEL

**Keywords:** floating-variant elbow dislocation, pediatric elbow dislocation, elbow dislocation, floating elbow, medial epicondyle fracture, medial elbow dislocation

## Abstract

Floating-variant elbow dislocation is a newly updated term used to describe several associations of fractures around the elbow and elbow dislocation that are still not adequately classified due to their rarity. Being extremely rare, only a handful of case reports are found in the literature describing this condition. Most of these papers described cases associated with the posterior or posterolateral direction of elbow dislocation, wherein each author has treated the fracture differently. The decision of surgical treatment, the order of fixation, the material used, and the need for ligamentous repair are all questions that are yet to be answered. We present herein a unique new variant of floating medial elbow dislocation in a 13-year-old female that was successfully treated by closed reduction of the elbow, open reduction of the distal humerus fracture, and orthopedic treatment of the radial shaft fracture.

## Introduction

Floating elbow is an uncommon injury to the elbow with a reported incidence of 2%-13% [1,2]. It was first mentioned by Stanitski and Micheli in 1980, where they described it as a dissociation of the pediatric elbow by the simultaneous occurrence of ipsilateral diaphyseal humerus fracture and forearm fracture [1]. Recently, this term was extended to include intra-articular or extra-articular distal humerus fractures, which are generally referred to as “variants” [2-5]. When the floating elbow is associated with a dislocation of the humeroulnar joint, the clinical scenario of floating elbow dislocation (FED) is even rarer. Several case reports have been described in the literature with the majority including posterolateral dislocation [3,5-10]. Herein, we present a rare case of medial dislocation of the elbow in the setting of floating elbow in a 13-year-old female. This case is a unique variant of FED since the type of dislocation is medial.

## Case presentation

**Figure 1:**
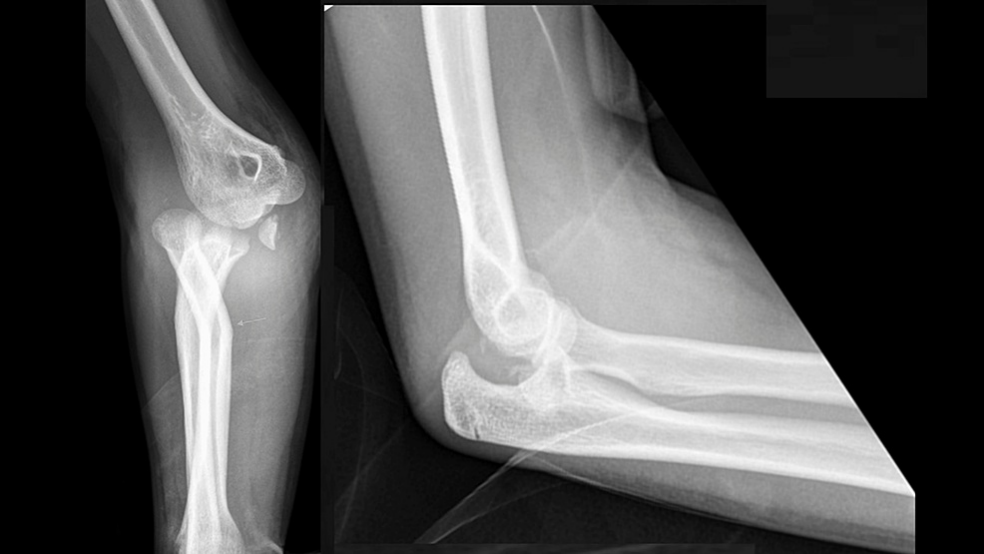
Radiographs of the elbow showing medial dislocation, medial epicondyle fracture, and non-displaced radial shaft fracture.

A 13-year-old female presented to the emergency department after sustaining a fall from a height of 3 meters, landing on her right hand while pronating her elbow. The patient had a deformation at her right elbow with severe pain. The neurovascular examination was unremarkable. A radiograph of the elbow showed a floating-variant medial dislocation of the elbow associated with medial epicondyle fracture and radial shaft fracture (Figure 1).

**Figure 2:**
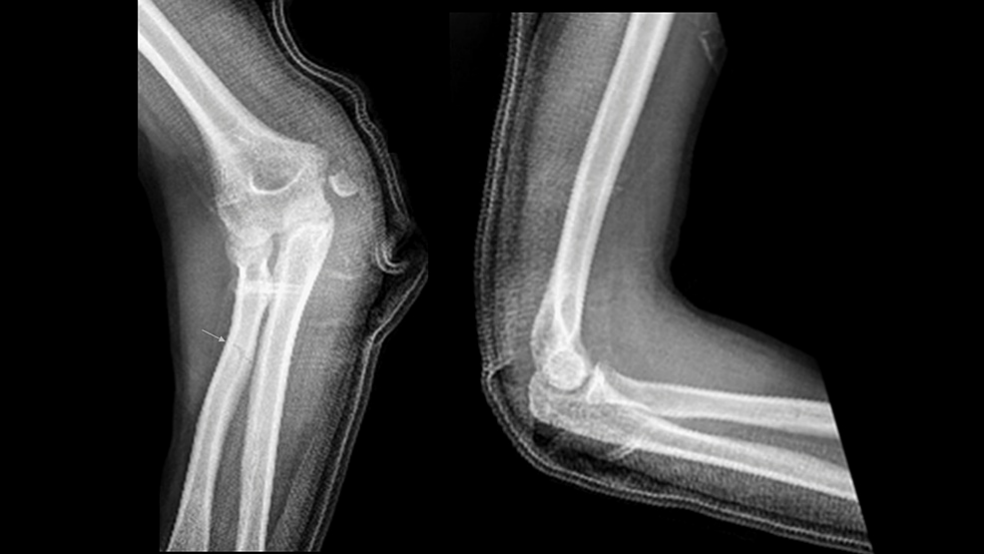
Radiographs of the elbow after elbow reduction showing displaced fracture of the medial epicondyle and non-displaced fracture of the radial shaft (arrow).

An urgent reduction was done in the emergency department with post-reduction radiographs shown in Figure 2.

**Figure 3:**
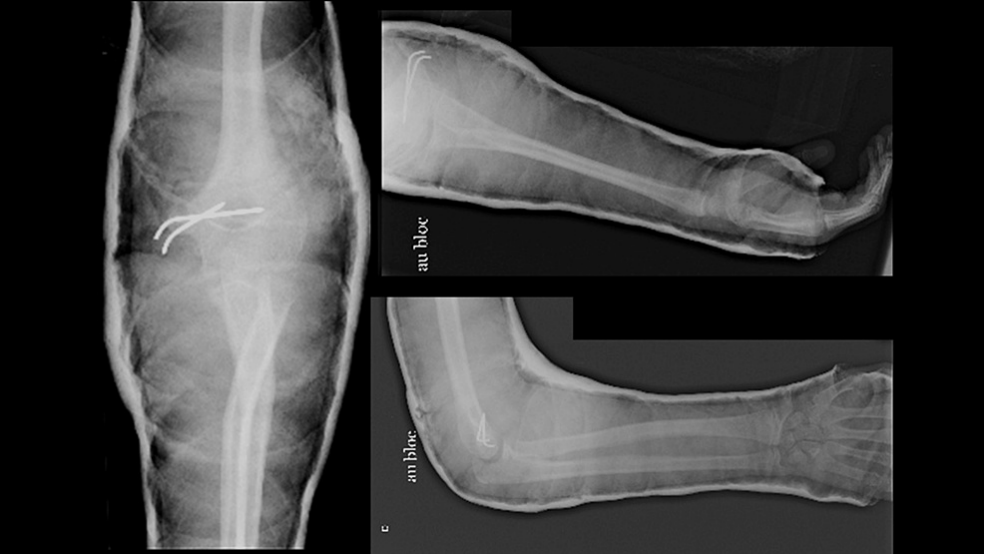
Radiographs of the elbow after open reduction internal fixation.

The patient was taken to the operating room where a medial approach to the elbow was done, followed by an open reduction internal fixation of the medial epicondyle. The epicondylar fragment was fixed using a 2-Kirschner wire. Testing of the elbow after reduction and fixation showed a stable arc of flexion and extension in addition to a stable valgus stress test but moderate laxity with a varus stress test. The radial shaft fracture was judged to be stable and non-displaced, so we did not approach it. A brachial antebrachial cast was then applied after closure. Postoperative radiographs are shown in Figure 3.

**Figure 4:**
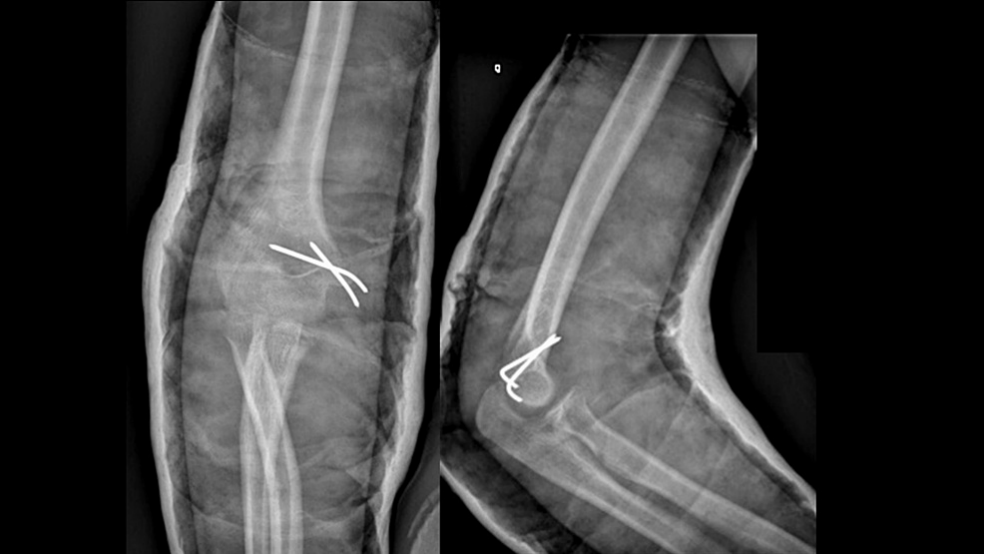
Radiographs of the elbow at the six-week follow-up showing a well-reduced elbow and stable fixation of the medial epicondyle.

At the six-week follow-up, the patient had no pain, and the radiographs showed good consolidation of the fracture lines. The cast was removed, and the patient was started on an auto-reeducation protocol where both active and passive range of motion were authorized as tolerated. Kirshner wires were removed six weeks post-operation (Figure 4).

**Figure 5:**
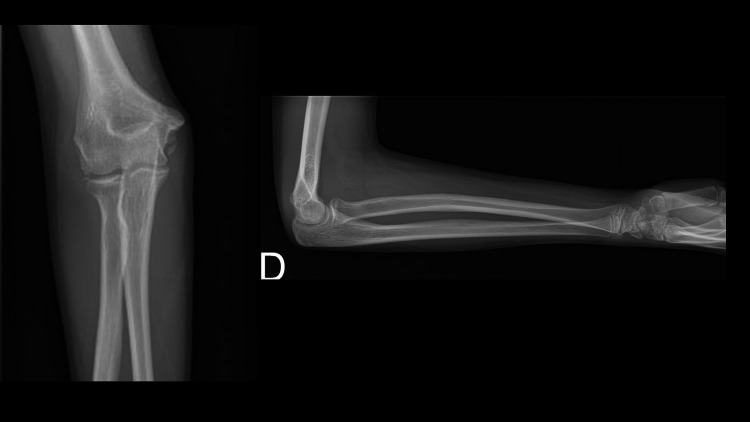
Radiographs of the elbow at the one-year follow-up.

At one year postoperatively, the patient was seen in the outpatient clinics, and her elbow was stable without any sign of stiffness or residual pain. Follow-up X-rays at that time showed a well-consolidated medial epicondyle and radial shaft, with some calcifications below the medial epicondyle, in addition to a well-centered elbow joint on both AP and lateral views (Figure 5).

## Discussion

FED is a very rare entity in elbow traumatology that is scarcely mentioned in the literature due to its association with two relatively infrequent pathologies: floating elbow with an incidence of 2%-13% [1] and elbow dislocation with an incidence of 3%-6% [11].

The first combination of these types of injuries was described by Viegas et al. [5], who reported a traditional floating elbow in a 57-year-old patient, combining a humeral shaft fracture with both bone forearm fractures. The type of dislocation found in this paper was the most frequent type of elbow dislocation, which is the posterolateral pattern. Similarly, Bettuzzi et al. [6] and Monreal [9] reported an identical pattern of injury in 2015 and 2018, respectively. The involvement of the articular surface in the fracture was first reported by Al-Zahrani [3], who described an articular fracture of the olecranon with both bone forearm fracture and humeral shaft fracture in the setting of elbow dislocation and radial nerve injury. Then, De Carli et al. [7] reported a new variant of FED associated with comminuted intra-articular distal humerus fracture. A third variant was described by Sarup and Bryant [10] and Bettuzzi et al. [6], who delineated an involvement of the proximal shoulder joint and distal radioulnar joint. Table 1 shows all reported cases of FED found in the literature.

**Table 1:** All reported cases of floating elbow dislocation found in the literature.

Author	Year of publication	Age	Mechanism of injury	Humeral component of floating elbow	Forearm component of floating elbow	Direction of dislocation	Order of reduction
Viegas et al. [5]	1989	59 years old	High-velocity injury	Midshaft humerus fracture	Both bone forearm fracture	Posterolateral dislocation	Elbow dislocation, forearm then humerus
Al-Zahrani [3]	1995	45 years old	High-velocity injury (motor vehicle accident)	Humeral shaft fracture	Both bone forearm fracture + olecranon fracture	Trans-olecranon dislocation	Humeral shaft, forearm shaft, intra-articular fracture (olecranon) then elbow dislocation
Sarup et al. [10]	1997	Adult age	High-velocity injury	Humeral shaft fracture	Galeazzi fracture	Posterior dislocation	Elbow dislocation, forearm then humerus
De Carli et al. [7]	2006	25 years old	High-velocity injury	Comminuted distal humerus fracture	Both bone forearm fracture	Posterior dislocation	Articular distal humerus (damage control due to soft tissue problem), elbow then forearm
Bettuzzi et al. [6]	2015	42 years old	Fall from a 3-meter height	Humeral shaft fracture + anterior shoulder dislocation	Both bone forearm fracture	Posterolateral dislocation	Humeral shaft, shoulder dislocation, forearm then elbow dislocation
El Ayoubi et al. [8]	2017	24 years old	Fall from a 4-meter height	Surgical neck and humeral shaft fracture	Distal radius fracture	Posterior dislocation	Elbow dislocation, humerus then distal radius
Monreal [9]	2018	24 years old	Fall from a 4-meter height	Midshaft humerus fracture	Both bone forearm fracture	Posterolateral dislocation	Forearm fracture, humerus fracture then elbow dislocation
Present case	2021	13 years old	Fall from a 3-meter height	Medial epicondylar fracture	Radial shaft fracture	Pure medial dislocation	Elbow dislocation followed by humeral epicondyle (forearm fracture was not displaced)

Remarkably, the direction of dislocation in all these reports was posterolateral dislocation, and we did not find a single paper that describes FED with a medial direction of elbow dislocation. Despite being simple, our case represents a unique pattern of FED where the forearm fracture is a radial shaft and the direction of dislocation is purely medial.

De Carli et al. [7] suggested a classification of floating dislocated elbow based on the involvement of the articular bone (type I: FED without articular bone involvement, type III: FED with articular bone involvement) and the involvement of wrist joint (type II).

After a careful literature review, we propose a modification to this classification to include all the reported patterns. Type I remains the same, including FED from shaft fracture without articular bone injury. Also, type II stays the same, including FED that are associated with the distal radioulnar joint. Then, we defined type III as FED from shaft fracture on one side and periarticular fracture on the other side. In this particular type, we cited type IIIA as FED resulting from shaft fracture on one side and articular distal humerus fracture (case reported by De Carli et al. [7]); type IIIB including the type described by Al-Zahrani [3], where the articular fracture is in the ulnar side [3]; and type IIIC, as in our case, where the distal humerus fracture is extra-articular and the pattern of fractures is relatively simple (non-displaced radial shaft fracture and displaced medial epicondyle fracture). Lastly, we will add type IV, involving dislocation of the shoulder joint (the case described by Bettuzzi et al. [6]). Table 2 shows our proposed classification.

**Table 2:** Proposed classification for floating elbow dislocation based on cases reported in the literature. [7]

Type	Modified De Carli classification of floating elbow dislocation (proposed by authors)
Type I	Floating dislocated elbow from shaft fractures without articular bone injury
Type II	Floating dislocated elbow with distal radioulnar joint dislocation
Type III	Floating dislocated elbow from shaft fracture on one side and periarticular fractures on the other side
	A: Floating dislocated elbow with intra-articular distal humerus fracture
	B: Floating dislocated elbow with intra-articular proximal ulnar fracture
	C: Floating dislocated elbow with extra-articular distal humerus fracture
Type IV	Floating dislocated elbow with shoulder dislocation

As a matter of fact, medial elbow dislocation represents one the rarest forms of elbow dislocations, and its occurrence, even in its simple pattern, was rarely mentioned in the literature [12-14].

In their retrospective study, Jockel et al. [12] found only four medial dislocations out of 184 elbow dislocations seen in their institute over an 11-year period. The analysis of their data found an en bloc avulsion of the lateral extensor origin along with the lateral collateral ligament at its humeral insertion in all patients. Consequently, they concluded that medial dislocation patterns may be associated with a higher rate of early instability and recurrence due to lateral ligamentous complex incompetence [12]. Our case was of a higher order of instability, where we had avulsion of the medial epicondyle along with all the medial ligamentous plane and flexor origin. Furthermore, our patient had a residual varus elbow instability even after fixation of the medial epicondyle where frank varus laxity was noticed on postoperative physical examination.

On the other hand, when a medial epicondyle fracture is seen, it is usually associated with posterolateral elbow dislocation. In 1977, Woods and Tullos studied the relationship between medial epicondyle fracture and instability. They defined “posterior and posterolateral dislocation” as an independent strict forward indication for surgical treatment of the medial epicondyle. This highlights the fact that posterior and posterolateral directions were the only type of dislocation found to be associated with medial epicondyle fracture [15]. Our case described a new variant where medial epicondyle fracture results from medially directed dislocation in a setting of a newly defined FED variant. We believe that this is a unique association that has never been seen in written literature.

The treatment of FED is not well established in the literature, where all reported cases were treated surgically. However, as we can clearly see in Table 1, there is no consensus on the order of reduction. Types I, II, and IV were all treated based on surgeon preference. In type IIIA and IIIB, FED involving articular fracture, reduction of the elbow fracture was performed only after the reduction and fixation of the articular fracture [5,7].

## Conclusions

Floating elbow dislocation is a chapter that is not well investigated in elbow traumatology due to the rarity of reported cases. We believe reporting more cases will allow more comprehensive classification that can ultimately lead to the development of treatment algorithms for this condition.

## References

[1] Stanitski CL, Micheli LJ (1980). Simultaneous ipsilateral fractures of the arm and forearm in children. Clin Orthop Relat Res.

[2] Moussa MK, Semaan D (2020). A pediatric floating elbow associating flexion type supracondylar fracture with both bone forearm fracture and ulnar nerve irritation: a case report. J Orthop Case Rep.

[3] Al-Zahrani SM (1995). Ipsilateral multiple injuries of the upper limb. Bahrain Med Bull.

[4] Mohamed SO, Ju W, Qin Y, Qi B (2019). The term "floating" used in traumatic orthopedics. Medicine (Baltimore).

[5] Viegas SF, Gogan W, Riley S (1989). Floating dislocated elbow: case report and review of the literature. J Trauma.

[6] Bettuzzi C, Cappuccio M, Cuoghi F, Tigani D (2015). Floating dislocated elbow with ipsilateral shoulder dislocation: double dislocation upper arm. J Orthop Sci.

[7] De Carli P, Boretto JG, Bourgeois WO, Gallucci GL (2006). Floating dislocated elbow: a variant with articular fracture of the humerus. J Trauma.

[8] El Ayoubi A, Maanouk R (2017). Floating dislocated elbow: a case report. Int J Bone Rheumatol Res.

[9] Monreal R (2018). Floating dislocated elbow in adults: a case report. Clin Res Orthop.

[10] Sarup S, Bryant PA (1997). Ipsilateral humeral shaft and Galeazzi fractures with a posterolateral dislocation of the elbow: a variant of the "floating dislocated elbow". J Trauma.

[11] Stans AA, Lawrence JT (2015). Dislocations of the elbow, medial epicondylar humerus fractures. Rockwood and Wilkins’ fractures in children, eighth edition.

[12] Jockel CR, Katolik LI, Zelouf DS (2013). Simple medial elbow dislocations: a rare injury at risk for early instability. J Hand Surg Am.

[13] Mehlhoff TL, Noble PC, Bennett JB, Tullos HS (1988). Simple dislocation of the elbow in the adult. Results after closed treatment. J Bone Joint Surg Am.

[14] Protzman RR (1978). Dislocation of the elbow joint. J Bone Joint Surg Am.

[15] Woods GW, Tullos HS (1977). Elbow instability and medial epicondyle fractures. Am J Sports Med.

